# Experts’ Views on Behaviour Change Techniques for Smoking Cessation in Pregnancy: A Qualitative Study

**DOI:** 10.3390/ijerph17217729

**Published:** 2020-10-22

**Authors:** Fizzah B. Abidi, Libby Laing, Sue Cooper, Tim Coleman, Katarzyna A. Campbell

**Affiliations:** Division of Primary Care, School of Medicine, University of Nottingham, Nottingham NG7 2RD, UK; mzyfba@nottingham.ac.uk (F.B.A.); libby.laing@nottingham.ac.uk (L.L.); sue.cooper@nottingham.ac.uk (S.C.); tim.coleman@nottingham.ac.uk (T.C.)

**Keywords:** smoking cessation, pregnancy, behaviour change, barriers and facilitators, qualitative analysis, expert group

## Abstract

Smoking during pregnancy is a global health problem which has devastating health implications. Behavioural support is an important part of smoking cessation support for pregnant women. Research has identified barriers and facilitators (B&Fs) and effective behaviour change techniques (BCTs) to aid women’s quit attempts. However, the extent to which and how these BCTs are used in practice is unclear. The research aimed to establish experts’ views on how behavioural support can be optimised and techniques operationalised in clinical practice, by identifying ways to address known B&Fs for smoking cessation in pregnancy. A focus group discussion took place with six experts, which highlighted how BCTs can be used in practice to support women in their quit attempts. A thematic analysis was conducted to elicit overarching themes. Five themes were found: involving the family, empowering women, using incentives to boost motivation, using practical techniques to help women with their quit attempts and managing expectations about nicotine replacement therapy. Empowering women to make their own decisions and encouraging small positive changes in smoking habits, using visual aids (e.g., growth charts) to inform women of the harms of smoking to the baby and treating families holistically were deemed important.

## 1. Introduction

The impact of smoking during pregnancy is an important health issue worldwide; it can be highly detrimental for both the mother and the baby. There are many risks, including developing pregnancy complications such as placental abruption, spontaneous abortions, ectopic pregnancies and placenta previa [1]. Additionally, there are increased likelihoods of babies being born with low birth weights or septal heart defects or later experiencing sudden infant death syndrome [1,2]. Later on in life, this is associated with a higher risk of them developing cardiovascular diseases and becoming smokers themselves [3,4]. Despite the risks, many women smoke during pregnancy today. It was reported that in the UK, 54% of women who are smokers try to quit at some point during the course of their pregnancy [5]. The most recent statistics for 2020/21 show that 9.8% of women were smoking at the time of their delivery in the UK [6]; this is higher than the national target stipulated by the Tobacco Control Plan for England of ≤ 6% by the end of 2022 [7]. Similar rates are seen in other developed countries. In the USA, it was reported that 7.1% of women were smoking during the time of their pregnancy in 2016 [8]. In Australia, 9.6% of women were smoking during pregnancy in 2018 [9]. This information highlights the importance of addressing this global health issue. 

Furthermore, certain factors have been found to influence the rates of smoking in pregnancy. Women who have support from family members or partners during a quit attempt have been found to be less likely to continue smoking during their pregnancy [10]. Studies also show correlation between socioeconomic background and the prevalence of smoking during pregnancy, where lower socioeconomic status is related to higher rates of smoking [8,9,11]. This information is supported by the findings of a systematic review which explored the barriers and facilitators (B&Fs) experienced by women who are trying to achieve smoking cessation [12]. The review found that disadvantaged smokers experience more barriers to smoking cessation than facilitators and that women’s relationships, including those with their partners and family members, may encourage or discourage quitting [12]. The smoking behaviour of pregnant women from disadvantaged backgrounds may also be influenced by access to tobacco. A recent study highlighted the correlation between higher tobacco store density and lower socioeconomic status with higher smoking-during-pregnancy rates [13]. Another study found predictors of maternal smoking during pregnancy related to women from lower socioeconomic backgrounds; these included unemployment, less time in education and women living alone [14]. Barriers related to psychological well-being were also identified as being highly influential; for example, women can perceive the stress related to a quit attempt to be more detrimental to their mental health and the health of the baby than continued smoking [12]. 

According to The National Institute of Health and Care Excellence (NICE) guidelines, in the UK, all pregnant women should be asked about their smoking and given a carbon monoxide (CO) monitoring test; smokers should be referred to stop smoking services (SSS) for behavioural support and followed up throughout pregnancy [15]. A longitudinal UK cohort study showed that despite showing interest when offered support, most women do not engage with it [16]. Women from socioeconomically disadvantaged backgrounds encounter the most barriers to achieving cessation, even after accessing support [17] and during the post-partum period [18]. Women who have a smoking and/or unsupportive partner also experience more barriers to quitting when pregnant [19]. Behavioural support is a key component of the interventions provided by SSS, and can involve techniques such as cognitive behavioural therapy, motivational interviewing or structured self-help [15], and evidence has shown that it is effective in aiding pregnant women to stop smoking [11,20]. Effective behaviour change interventions should systemically address key B&Fs for behaviour change by catering to an individual’s physical, social and emotional needs [21]. These interventions can be delivered in a group or on an individual basis, either face to face or over the telephone, with a specialist advisor [22]. Recent research considered smoking cessation experts’ views on which B&Fs should be addressed as a priority, when supporting pregnant women in their quit attempts [23,24]. In these studies, the experts identified smoking being a norm in women’s social circles and a lack of support from partners to be the key barriers to successfully quitting whilst pregnant. Stopping smoking not being perceived as a priority by the women, a lack of motivation and low self-belief in their ability to quit were also noted as being difficult to help women to overcome [23,24]. 

Behavioural change techniques (BCTs) are the smallest active components of a behaviour change intervention, which have the potential to change a behaviour, either in isolation or in combination with other BCTs [25]. Examples of BCTs include goal setting, action planning, feedback and monitoring or behavioural substitution [26]. They are known as “replicable” components of an intervention that aim to “redirect causal processes that regulate behaviour” [27] (p. 2). Previous studies explored the BCT contents of effective interventions for smoking cessation in pregnancy [20,24] and found that BCTs such as information provision, goal setting, social support and rewards contingent on successfully stopping smoking were most commonly found in effective interventions [20,24]. However, Lorencatto and colleagues also highlighted that in practice, only a limited number of effective techniques are used by SSS [20]. Smoking cessation experts also noted that these BCTs are often inconsistently operationalised [23,24].

The study reported here is a part of a larger body of research into how behavioural smoking cessation support can be developed to improve quitting outcomes [23,24]. The aims of this study were to gain an insight into how smoking cessation experts operationalise or put to use previously identified effective BCTs in routine practice [23,24].

## 2. Materials and Methods

Data were collected in a focus group meeting with UK-based experts in smoking cessation in pregnancy, on the 15 November 2017. Ethical approval was granted by the East of Scotland Research Ethics Service REC 1, REC reference number 16/ES/0125, on the 22 September 2016. 

### 2.1. Recruitment

Health professionals, including smoking cessation and antenatal care specialists, with experience in the field of smoking cessation in pregnancy were considered “experts” [28]. Participants were eligible if they were over the age of 18, spoke English and could give informed consent. 

Potential participants were identified through the National Centre for Smoking Cessation and Training (NCSCT), from contacts from previous studies and by using snowballing methods. Eighteen smoking cessation and antenatal leads were approached via email in September 2017. They received a description of the study, a consent form and an information sheet providing details of this research and participants’ rights. A reminder was sent one week later. Those who expressed interest were contacted again to arrange a date for the meeting. As a result, eight experts completed a Doodle Poll (an online meeting organising software); seven were available on the same day.

### 2.2. Focus Group Meeting

The meeting was facilitated by two convenors: K.A.C. (author, the study lead involved in recruitment and who had established a professional relationship with some participants) and J.D. (an independent convenor, to reduce interviewer bias). The meeting lasted four hours and was conducted in three sessions. The participants were asked to respect the confidentiality of the information exchanged and were informed the data would be anonymised to facilitate open and honest discussion. 

The convenors welcomed the participants and informed them of what the meeting would entail. The participants were asked to read lists of barriers and facilitators (B&Fs) for smoking cessation in pregnancy and techniques that addressed these (derived from previous research [23,24]). These B&Fs and techniques were grouped into Theoretical Domains Framework (TDF) domains prior to the meeting [24,29]. The TDF is an integrative framework that can help to identify key B&Fs for behaviour change and assist with mapping appropriate BCTs to those B&Fs through 14 life domains. The key domains used in this study were social influences, knowledge, intentions, beliefs about capabilities and emotions [29]. The TDF has been used successfully in designing behavioural smoking cessation interventions [30], and was adopted in this study as it can aid in bridging the gap between research and evidence-based practice to improve behavioural support for pregnant smokers.

The participants were asked to provide and discuss examples, based on their best clinical practice and experience, of how these techniques can be effectively operationalised. The facilitators ensured that the meeting was running to time, that everyone contributed, and that the discussion was staying on topic.

The meeting was audio-recorded and transcribed using external transcription services.

### 2.3. Analysis

An inductive thematic analysis was conducted [31] and aimed to pick out the themes which best recognised ways of operationalising previously identified techniques [23]. An inductive approach allows the themes to be formulated based on the information provided by the data. This method of analysing data was chosen as it allowed us to learn from the experiences of the experts and synthesise information from a large data set [31]. 

One author (Fizzah B. Abidi) read the transcripts multiple times to identify ideas and concepts that related to the aims of the study; these initial codes were then grouped into themes, which were identified at the semantic level of the data [31]. Mind maps were used to narrow down the themes by merging similar codes, highlight links and identify overlapping themes and sub-themes within the broader themes [31]. To confirm the relevance of the themes, the data were re-coded using these themes and sub-themes. At this stage, two authors (Katarzyna A. Campbell and Libby Laing) also coded the data, to ensure the validity of the process. The second coders looked at 50% of the data, stated the agreement with the existing codes and offered comments and suggestions to improve or clarify the coding framework. The coding framework was then finalised; quotes and detailed descriptions for each theme and sub-theme were included in a table to ensure that every example of the theme would be identified during the coding process. The data were then coded for a final time. 

### 2.4. Reflexive Statement

This study was conducted from the critical realism stance, which assumes that our understanding of reality is mediated by our beliefs and perceptions [32]. We note, therefore, that it is important to consider the characteristics, beliefs and experiences of the authors and how these could have affected the data collection and analysis. Katarzyna A. Campbell, Libby Laing, Sue Cooper and Tim Coleman are researchers with backgrounds in primary care, public health and/or health psychology, with extensive experience in conducting qualitative research and research in the field of smoking cessation in pregnancy. Fizzah B. Abidi conducted data analysis, and at the time of the study, she was a BMedSci Honours student with interest in epidemiology, public health and preventative approaches to global health issues. None were smokers. The study was conducted in the UK, where smoking in pregnancy is a considerable health problem, and smoking cessation, including NRT, was available to all pregnant women, free of charge, at the time of data collection. At the onset of the study, all authors considered smoking cessation in antenatal care to be one of key health priorities and considered NRT a valuable tool for aiding smoking cessation. The authors remained aware of their views throughout the process of data collection and analysis and attempted to ensure that all data were treated equally and that contradictory findings were given equal place.

## 3. Results

Out of the seven experts who completed the Doodle poll, six experts were able to take part in the meeting on the day. The participants had experience working across smoking cessation settings and antenatal settings, including antenatal clinics, community midwifery and working as smoking cessation advisors in primary and secondary care and community venues. The numbers of years of experience in a relevant role ranged from 3 to 17 years. Details about the area of work of each participant, how many years of experience they have and the geographical location of where they work is summarised in Table 1.

Five themes were identified. These were empowering women, involving the family, practical techniques to help women with quit attempts, using incentives to boost motivation and managing expectations of NRT. Table 2 shows a summary of these themes and the sub-themes. 

### 3.1. Empowering Women

#### 3.1.1. Empowering Women to Make Their Own Decisions

The participants reported that giving women the relevant information and/or support for them to make their own decisions, where they do not feel forced, is influential.

“…it’s more about…helping people come to those conclusions rather than ramming it down their throat I think.”, P5.

“…we can talk about what those risks are and they start to put two and two together (…) we find that the women come to the conclusion themselves (…) we get a lot more success if it has come from them.”, P2.

#### 3.1.2. Empowering and Supporting Women Who Are Not Ready to Quit

Many women were reported to find committing to a full quit attempt difficult. The participants empowered such women by encouraging them to take small steps to make positive changes in their smoking habits. This was seen to be beneficial, as the women *“feel better about themselves”* (P2). Small steps reported by the participants were encouraging them to come to the next appointment, even if there was no quit date, and encouraging a reduction in smoking. 

“…we need to set short term goals for a long-term gain…sometimes, people smoke in their bedrooms …I will say to them… ‘for this week, that’s our goal is that you don’t smoke in the bedroom’”, P1.

However, some women may *“decide it isn’t their time to change*” (P1); they should be given the option to resume contact once they do feel ready: “*I say to them…’here’s my number if anything changes just let me know”* (P1). In instances where other ongoing issues in a woman’s life may be the hindering her quit attempt, *“signposting to other services…counselling…”* (P3) were thought to be important factors to consider. 

### 3.2. Involving the Family

#### 3.2.1. Holistic Approach to Treating the Family Unit

The participants highlighted the importance of supporting a family holistically by giving examples of supporting other smokers in the family to quit alongside the pregnant women and by encouraging family members to play an active role in the quit attempt of the pregnant woman. Inviting family members to appointments, involving them in the conversation during home visits and addressing misinformation that came from family members and their emotions were discussed within the group.

“…certainly, assessing everyone else who smokes to support the rest of the family and make it a familial thing and not treat them in isolation definitely, definitely works.”, P1.

The participants noted that sometimes, family members, for example, the pregnant woman’s mother, may try to diminish the risk of smoking in pregnancy by saying they smoked throughout their pregnancy and their children turned out fine. They discussed the potential sense of “internal guilt” *(P5)* these family members may be experiencing and emphasised the importance of supporting these family members to alleviate this guilt and turn it into a positive lesson that could help them support the pregnant woman’s quit attempt more successfully.

“…there’s always the clever person who’s been there often, a mum or a dad or grandma who’s ‘well I’ve smoked all my life and I’ve had six kids and they’re fine’ (…) I never, ever disagree with the person and I say ‘that’s fine and you were lucky, your children are fine, however, we’ve moved on a bit since then and we now know that a percentage of children that either are born prematurely or sadly they don’t make it (…) we know that if you smoke and your daughter smokes, your children are likely to smoke as well and my job is to try to make smoking history for children.”, P1.

#### 3.2.2. Promoting Smoke-Free Environments

While it was clear that it is most beneficial for all family members to quit smoking, the participants sympathised that not all family members of a pregnant woman would be willing to quit with her. Achieving smoke-free environments, where all smokers smoke outside, away from the woman and the baby, and protect them from second- and third-hand smoke, was considered an acceptable compromise. This required the women and their family members to understand the risks of second-hand smoke and build strategies to establish a smoke-free environment. The participants highlighted the need to encourage women to have open communication with family members to achieve this. 

“…make them realise the risks of it because they often think it doesn’t matter, ‘my mother’s been this morning, I couldn’t ask her to go seven steps out could I?’, ’well actually yes you can’…”, P1.

### 3.3. Practical Techniques to Help Women with Quit Attempts

#### 3.3.1. Using Visual Aids with Clear Explanations to Improve Motivation to Quit in Pregnancy and/or Compliance with NRT

The participants highlighted the importance of visual aids with clear explanations as a technique to help women to understand the risks of smoking to their baby, understand the science behind addiction and improve compliance with NRT. Examples of the visual aids reported were carbon monoxide (CO) monitoring, growth charts, scans and pictures of the brain. 

“…what works particularly well is the visual aid, letting the woman interact…in something which reinforces that message…it tells her…we can prove that that’s happening…to your baby”, P1.

The participants reported that CO monitoring is useful, as “showing the woman the reading that is the baby’s foetal carbon monoxide hemoglobin means something to them (…) they understand then that their smoking is affecting their baby” (P1). Additionally, it was reported that CO monitoring can improve motivation, as women “like to see they’re improving, they like to see they’re doing the best for their babies” (P1).

Growth charts and scans with an explanation were identified by the participants as methods of showing the effects of smoking on their babies’ growth. 

“…the customised growth charts and that is a fantastic incentive to women because they can see that their baby has dropped off its line and that is scary…”, P1.

“we do serial scans for smoking ladies because…her baby had stopped growing, she was worried about it. I went to see her (pregnant woman and her mother) the two ladies quit together, and the pregnant mum went on to have a baby of about 6 lbs 8, which it was not estimated to be”, P1.

The participants found brain pictures useful for explaining the science behind addiction and withdrawal symptoms. These were diagrams that could be used to educate women about the reward pathway and the roles of dopamine and receptors. The participants highlighted that this also helped women understand the importance of using NRT, and they therefore thought it improved compliance.

“…something that we’ve been using that works really, really well with our advisors is that sort of brain picture which helps to visually educate the woman…actually the cigarettes are causing the stress in the first place and women often say ‘I never understood that. I never saw it from that point of view ‘…which just gets away from all of that sort of, you know, the problems with complying with NRT, managing withdrawal symptoms and the stress as well”, P4.

#### 3.3.2. Helping Women Develop Coping Strategies to Deal with Negative Emotions

The participants felt many women go through negative emotions such as guilt, anger and stress during a quit attempt and highlighted the importance of equipping women with relevant coping strategies to overcome this challenge. 

The examples reported included turning “negative emotions…into positives…trying to help them remove their guilt and use it for positive reasons” and “…re-channelling that…anger…in the right direction” (P6). Another participant described that “using mindfulness techniques…dealing with the here and now…acknowledging what’s happened” (P2) could be used as a strategy. 

#### 3.3.3. Helping Women Develop Substitute Behaviours to Avoid Smoking

The participants highlighted the importance of advising women to use substitute behaviours to avoid smoking. The examples reported by the participants include behavioural substitutes (such as breathing techniques) and food substitutes, and some examples of personalised substitutes were given too (such as chewing straws). 

“…have a list of different things you could do…some stop smoking services used to give out stress balls and hand balls…breathing techniques…from lots of practical things and just random wild things”, P6. 

### 3.4. Using Incentives to Boost Motivation

#### 3.4.1. Rewards

The participants reported examples of using rewards to boost women’s motivation to quit smoking. Incentive schemes were one of the reported methods of offering rewards to women. 

“…I found that beneficial in terms of…recognition and reward and that praise…It was just a good opportunity to help them stay quit rather than maybe to get them to quit for that £15”, P3.

It was reported that *“incremental”* incentive schemes were seen as more impactful, as women *“can reflect back”* (P6) on how much money they have accumulated through the scheme. 

Apps that count how long someone has stayed smoke-free for or how much money someone has saved since quitting smoking were reported to be rewarding for some women. 

“…one lady, she used one of the free apps so she were able to see how much she was saving…she’s still quit now, I still keep in contact with her and she’s kept it running…if she struggles she’ll look back…that’s keeping her going”, P3.

Encouraging self-reward was another method reported to boost motivation; examples given by the participants included holidays, saving money and items for the woman herself. 

“I often say ‘buy yourself one of those little tins that you can’t take out of and even if you put £5 a day in it, which is only half your smoking budget then in a month’…‘you’ll have this much money and treat yourself’…I will say ‘treat yourself…shoes or boots or a top…even if you just get some bath bombs and go and lie in the bath’”, P1.

#### 3.4.2. Verbal Praise

The participants highlighted the importance of offering women verbal praise to boost their motivation.

“…look at the positive things, even if their longest quit attempt was three weeks, ‘you can do it for three weeks, you’ve already done that, you’ve already got it within you’”, P5.

### 3.5. Managing Expectations of NRT

It was reported that managing women’s expectations of the role of NRT during the quit attempt was thought to be useful. Stating the timeframe needed for it to work and the negative aspects of using the therapy were reported to improve compliance. 

“…they know it’s not a magic solution, a magic patch, there is a lot of work as well that needs to go alongside it, so it’s managing those expectations”, P3.

“…it can taste pretty disgusting to start with but if you use it properly and for long enough, you will even grow to like it. And then women go away thinking actually, yeah, she said it would taste like this, but she said that I need to carry on”, P4. 

## 4. Discussion

### 4.1. Key Findings

This study reports how smoking cessation experts operationalise previously identified effective BCTs in routine practice. There were three key findings. Firstly, empowering and supporting women to make their own decisions and including women who are not ready to quit were thought to be important. Secondly, the experts also emphasised the importance of family involvement during the quitting process. Finally, practical techniques that aided quit attempts were also identified by experts; these included visual teaching aids with clear explanations, which mainly helped women in understanding the harms of smoking, helping women to identify effective coping strategies to deal with negative emotions, and substitute behaviours. 

### 4.2. Strengths and Weaknesses

There were some limitations to this study. Firstly, this meeting took place among only one small group consisting of six experts; this meant that some themes were derived from only two to three experts’ experiences. Conversely, the small group size allowed for an in-depth discussion and gave each participant ample time to present their experiences. Moreover, four of the experts had experience in working in the East Midlands region of the UK, so their experiences were likely to have been similar. In the UK, the support provided by SSS varies depending on the region [33], which may not have been represented in these data. These factors pose a limitation for the generalizability of the findings to a broader context. However, the group consisted of experts who had varying years of experience and different backgrounds in the field, which offered a variety of experiences to counteract this potential risk. 

Additionally, the meeting was conducted in a group setting. This may have meant experts did not feel comfortable disclosing certain experiences. This could include experiences where women had not responded well to a specific technique they used because they could have felt this reflected on their own ability, or experiences which involved sensitive or confidential information. However, as it was outlined to the group by the convenors before the discussion began that the researchers were confined to confidentiality, that the transcripts would be anonymised and that everyone in the room should respect the information shared in a confidential manner, this would have helped to overcome any potential reservations experts had when it came to disclosing information. 

On the other hand, a strength of this study is that it used a face-to-face expert group meeting to collect data; the interaction between the participants was able to encourage active discussion of the useful techniques, and the views provided by each participant could be validated or countered by other experts’ experiences [34]. Allowing the experts to hear about each other’s experiences could have prompted them to share their own that were related which could otherwise have been forgotten if they were being interviewed alone.

Additionally, the meeting was structured into domains, and the group convenors ensured each one was discussed, meaning that the discussion covered a variety of different barriers and BCTs. This was beneficial, as the experts were able to consider different aspects of behavioural support, which was reflected in the findings of the study. 

### 4.3. Discussion in Context of Previous Literature

Empowering women to make their own decisions relating to their quit attempt was a key message highlighted in this study. Supporting women’s thought processes with well-assimilated information and allowing them to feel in control of their decision resulted in more successful outcomes, in comparison to when they feel pressurised to do so. This is supported by the theory of shared decision making (SDM) [35]; 2019 NICE guidance states that the healthcare professional and patient should work together to reach a decision and patients should be well-informed and feel empowered in order to reach a decision; this guidance is appropriate across different healthcare settings and can be applied to smoking cessation [36].

Statistics show that approximately 50% of women who smoke quit during their pregnancy [5]. Previous research has concluded that it is important to identify why certain women fail to engage with smoking cessation services whilst pregnant [37]. This study found that some women may not be ready to quit smoking as other factors may be hindering them such as issues surrounding mental well-being [23,38]. Similarly, in a previous study, experts thought “quitting being low on women’s list of priorities” was a barrier to smoking cessation that was difficult to address with BCTs [24], so women who are experiencing other life problems may not prioritise their quit attempt. Signposting these women to the relevant support services may aid them to eventually quit smoking. The participants in the current study also identified that empowering women by encouraging small positive changes including a reduction in smoking can be effective for this group. This is supported by a qualitative study which found that cutting down can increase the chances of a permanent quit attempt [39]. However, current NICE guidelines suggest that health professionals should encourage quitting rather than cutting down [15]. Future research that informs guidelines should consider the prospect of a reduction in smoking for women with more profound circumstances where a quit attempt straight away may not be a feasible option. 

Smoking being a social norm and a lack of family support have been identified as barriers to pregnant women quitting smoking in previous research [12,24]. The participants in this study felt that the influence of social networks, particularly the partners and mothers of the pregnant women, was an important factor to address when supporting women to become smoke-free. Using a holistic approach to treat the family unit was identified as potentially helpful in women’s quit attempts, which builds on the findings of a previous study which suggested that encouraging partners to quit and involving them in the treatment process were appropriate techniques to help support women [23]. This study reported useful methods for operationalising these techniques such as inviting family members to appointments, addressing misinformation that women receive from their family members and working with families to create smoke-free homes. This could help form the basis of enabling the implementation of this research into clinical practice, which is important, as a previous study has shown that BCTs addressing social support are important and need development [20,24].

This study found that using visual aids to help educate women about addiction and the harms of smoking during pregnancy to the baby could aid their understanding of these concepts and optimise smoking cessation support. Previously, a recent multi-centred, community-based participatory research project was carried out to elicit the views of healthcare professionals on a resource package developed to support Aboriginal pregnant women to quit smoking during pregnancy [40]. The need for more visual aids to encourage women to engage in conversation about their smoking and to put across the right message was highlighted [40]. A similar theme emerged in this study, where visual aids such as growth charts and serial scans enabled women to see and therefore understand the harm smoking was having on the growth of their babies. This technique is also pregnancy specific, building on an important facilitator of smoking cessation in pregnancy, “women’s desire to protect their children”. This is valuable, as the need to tailor support such that it is specific to pregnant women was highlighted in another previous study [24]. Additionally, CO monitoring was found in this study by experts to have a positive influence on pregnant women’s smoking behaviours. It was also found that it is crucial to accompany visual aids with clear explanations. The findings of a qualitative study evaluating pregnant women’s views on an “Opt Out” referral pathway found that while some women felt CO monitoring was useful for understanding the harms of smoking to their baby, other women felt that the numbers had less impact on their motivation to quit, as the significance of them had not been explained [41].

Additionally, it was found that visual aids such as brain pictures/diagrams can be used to aid women’s understanding of addiction. In a recent study, experts identified a lack of this understanding to be a barrier to smoking cessation [23]. Another study where women’s views about NRT were elicited found that women had concerns about increasing their nicotine addiction, causing a lack of adherence to the treatment [42]. In this study, brain pictures with clear explanations were found to be helpful when explaining to women how nicotine causes addiction and why they experience withdrawal symptoms. This understanding can in turn improve compliance with NRT, as it can overcome women’s concerns with regard to NRT causing further nicotine addiction, which has been proven to be untrue [43]. 

Previous research has identified that women often feel they are not given enough practical advice on how to become smoke-free [12]. This study recognised practical methods that health professionals could recommend to women to potentially aid their quit attempts. The examples reported were using substitute behaviours, coping strategies, self-reward and recommendations of how to cut down smoking, where this approach was appropriate. The importance of coping strategies is supported by existing research [11]. 

Using incentives, including rewards and praise, was identified as being able to boost women’s motivation during a quit attempt. The concept of saving money was a reward for some women. This is confirmed by recent findings from another study where the participants reached agreement that “explaining financial benefits to quitting” aids women to stop smoking [23]. Incentive schemes were also seen to have a positive influence on women’s motivation, which is also supported by findings in another study [11]. Furthermore, women from disadvantaged backgrounds have lower success rates for quitting smoking during pregnancy [5,17,38]. Many women globally are affected by health disparities, which cause increased rates of smoking during pregnancy in women from lower socioeconomic groups [13,14], so it is important to ensure smoking cessation interventions continue to develop methods of engaging this group of women. Incentive schemes have also previously been reported to be useful in this group of women [44]. Praise was also identified as an incentive for women to improve their motivation, which is supported by previous findings [23]. This study builds on existing knowledge by highlighting how women can be rewarded in real life, such as using apps, self-rewards or incremental incentive schemes.

### 4.4. Further Work

This study builds on previous findings, exploring practical ways to operationalise frequently used BCTs [15,17,19]. While the current study offers some valuable insights into the practical use of BCTs, further research is required to establish a consensus and build upon these methods to optimise support. Research involving more experts would be invaluable to improve the generalisability of these findings. Additionally, it would be beneficial to gather the opinions of pregnant women who smoke on these approaches, as they can provide novel insight into how support can be further optimised. 

Furthermore, research into the feasibility of incorporating encouraging a reduction in smoking for women who are not ready to quit smoking completely would be useful to inform future guidelines for smoking cessation support. A study which compares this approach with the current NICE guidelines, which emphasise the encouragement of full quit attempts [15], would be useful.

Additionally, practical techniques and visual aids with clear explanations, which were identified in this study as themes, can form the basis of future research. Quantitative research would be valuable to build on these and confirm if these approaches are effective, so that they can be more widely applied in practice. 

## 5. Conclusions

Empowering women by providing them with relevant information to make decisions and involving them in the decision-making process was perceived by the experts to be an important way of providing behavioural support. Furthermore, it was also reported that in some cases, encouraging small positive changes in smoking habits such as reduction also empowered women, and as a result, women were more likely to quit. Additionally, involving the family was thought to be a key part of delivering behavioural support. Furthermore, the use of visual aids such as growth charts helped women to understand the harms of smoking to their babies, which was influential, as the well-being of their babies is an important factor in motivating them to quit. 

## Figures and Tables

**Table 1:** Summary of relevant details of the participants of the expert group.

Participant ID	Area of Work	Years of Experience	Location of Work
P1	Smoking cessation and antenatal care	35	North West
P2	Antenatal care and smoking cessation champion	15	East Midlands
P3	Smoking cessation	3	East Midlands
P4	Smoking cessation and antenatal care	17	Yorkshire and Humber; Nationwide
P5	Smoking cessation	13	East Midlands
P6	Smoking cessation	9	East Midlands

**Table 2:** Summary of the themes and sub-themes.

Theme	Sub-Themes
Empowering women	1.1. Empowering women to make their own decision
	1.2. Empowering and supporting women who are not ready to quit
2. Involving the family	2.1. Holistic approach to treating the family unit
	2.2. Promoting smoke-free environments
3. Practical techniques to help women with quit attempts	3.1. Using visual aids with clear explanations to improve motivation to quit in pregnancy and/or compliance with NRT
	3.2. Helping women develop coping strategies to deal with negative emotions
	3.3. Helping women develop substitute behaviours to avoid smoking
4. Using incentives to boost motivation	4.1. Rewards
	4.2. Verbal praise
5. Managing expectations of NRT	
